# Shaping the Future of Perinatal Cells: Lessons From the Past and Interpretations of the Present

**DOI:** 10.3389/fbioe.2019.00075

**Published:** 2019-04-10

**Authors:** Antonietta R. Silini, Alice Masserdotti, Andrea Papait, Ornella Parolini

**Affiliations:** ^1^Centro di Ricerca E. Menni, Fondazione Poliambulanza, Brescia, Italy; ^2^Istituto di Anatomia Umana e Biologia Cellulare, Università Cattolica del Sacro Cuore, Rome, Italy

**Keywords:** regenerative medicine, perinatal tissues, human term placenta, amniotic membrane, immunomodulation

## Abstract

Since their discovery and characterization, mesenchymal stromal cells (MSC) have been a topic of great interest in regenerative medicine. Over the last 10 years, detailed studies investigated the properties of MSC from perinatal tissues and have indicated that these cells may represent important tools for restoring tissue damage or promoting regeneration and repair of the tissue microenvironment. At first, perinatal tissue-derived MSC drew attention due to their potential differentiation capacities suggested by their early embryological origin. It is nowadays accepted that perinatal tissue-derived MSC are promising for a wide range of regenerative medicine applications because of their unique immune modulatory properties, rather than their differentiation ability. As a matter of fact, the activation and function of various cells of the innate and adaptive immune systems are suppressed and modulated by MSC from different perinatal tissues, such as human term placenta. However, the mechanisms by which they act on immune cells to facilitate tissue repair during pathological processes remain to be thoroughly elucidated to develop safe and efficient therapeutic approaches. In addition to immune modulatory ability, several other peculiar characteristics of placenta MSC, less explored and/or more debated, are being investigated. These include an understanding of the anti-microbial properties and the role of placental MSC in tumor progression. Moreover, a thorough investigation on preparation methods, bioactive factors, mechanisms of action of the cell secretome, and the development of potency assays to predict clinical efficacy of placenta MSC and their products, are necessary to provide a solid basis for their clinical application.

## Lessons from the Past

The advent of cell therapies has offered promising therapeutic options especially for degenerative and inflammatory diseases. Amongst the cell populations that initiated this promise are mesenchymal stromal cells (MSC). One of the most intriguing sources of MSC that has attracted much attention in the past decade are birth-associated tissues, or perinatal tissues, such as the human term placenta. Nonetheless, the rise to fame for placenta-derived MSC has been turbulent, with many demands to meet, especially since they were born/discovered in the era when bone marrow-derived and adipose tissue-derived MSC were galloping contenders.

### Back to the Basics: Rationale for Placenta as a Cell Source

Placental MSC were identified many years ago and their existence has been known for some time. However, the first demonstration of their potential application in cell therapy was in 2004 and our lab demonstrated that MSC from fetal membranes can be transplanted without signs of immunological rejection (Bailo et al., 2004). In an editorial by Heeger (2004), a play on words underlined the infancy of the field at that time, and highlighted the interest of the transplantation community in keeping updated on the bright future of placental tissues in transplantation (Heeger, 2004).

The rationale for our first idea to investigate placenta for its potential in regenerative medicine is summarized as two pillars of cell transplantation: one representing stem cell differentiation potential and the other representing lack of rejection. Cells derived from the placenta could meet these two features as their early embryological origin could favor the hypothesis of stem-cell potential (Parolini and Soncini, 2006), and the fact that the placenta contributes to the development and growth of a semiallogeneic fetus during pregnancy favors the idea that cells from the placenta could possess some intrinsic, peculiar immunological characteristics.

In addition, more practical and logistical reasons made the placenta an attractive cell source. It is easily obtained after birth without invasive procedures, and it is considered biological waste thus bypassing ethical tensions associated to other cell sources.

### Placenta Tissues and Placenta-Derived Cells

The human term placenta is a unique organ comprised of different tissues, including maternal (decidua) and fetal tissues. According to the First International Workshop on Placenta-Derived Stem Cells held in Brescia, Italy in 2007 (Parolini et al., 2008), four major regions of fetal placenta, thought to harbor potential stem/progenitor cells, were identified: amniotic epithelial, amniotic mesenchymal, chorionic mesenchymal, and chorionic trophoblastic tissues. A consensus was reached according to which at least four different cell populations have been distinguished: human amniotic epithelial cells, human amniotic MSC, human chorionic MSC, and human chorionic trophoblastic cells (Bailo et al., 2004; In't Anker et al., 2004; Ilancheran et al., 2007; Soncini et al., 2007; Parolini et al., 2008). Cells with MSC properties have also been isolated from other placental tissues, such as the chorionic villi (Fukuchi et al., 2004; Igura et al., 2004; Portmann-Lanz et al., 2006; Castrechini et al., 2010; Abumaree et al., 2013; Roselli et al., 2013), the maternal decidua basalis (In't Anker et al., 2004; Macias et al., 2010; Abomaray et al., 2016), and from different compartments of the umbilical cord, such as the Wharton's jelly (Troyer and Weiss, 2008; La Rocca et al., 2009a;La Rocca et al., 2009b).

Herein we will focus on MSC from the amniotic membrane (hAMSC) which has been the main topic of our laboratory for almost 2 decades.

### Placenta MSC Differentiation

One of the first questions raised was whether or not placental MSC fulfilled the International Society for Cell and Gene Therapy (ISCT) minimal criteria for MSC, that is, the adherence to plastic, specific cell phenotype, and tri-lineage differentiation potential toward osteoblasts, adipocytes, and chondroblasts using standard *in vitro* differentiating conditions (Dominici et al., 2006). As placenta-derived MSC were being intensely investigated, a consensus specific to placenta, and more specifically to MSC from amniotic membrane and the chorionic mesenchymal and chorionic trophoblast regions, was established in 2008 (Parolini et al., 2008). For the most part the minimal criteria were common to those established by ISCT, with the exception of the fetal origin of amniotic MSC and of more lenient criteria for their differentiation capabilities. As a matter of fact, the consensus stated that amniotic and chorionic MSC should demonstrate *in vitro* differentiation potential toward at least one lineage, including osteogenic, adipogenic, chondrogenic, and vascular/endothelial (Parolini et al., 2008). Evidence has now demonstrated that placental MSC, and especially amniotic membrane-derived MSC (hAMSC), are not front runners for in vitro cell differentiation (Wegmeyer et al., 2013; Kmiecik et al., 2015; Wu et al., 2018). In addition, the *in vivo* differentiation potential of hAMSC remains obscure.

### Immune Modulatory Properties: The Claim to Fame for Placenta MSC

At the time hAMSC were discovered (Bailo et al., 2004; Soncini et al., 2007), their counterparts from bone marrow had already been acknowledged as suppressors of T cell proliferation (Bartholomew et al., 2002; Di Nicola et al., 2002). These initial studies, along with the hypothesis that the placenta could harbor cells with intrinsic immunological properties due to the unique immunological setting during gestation, redirected the attention from the differentiation capacities of placental MSC toward their potential regulatory effects on immune cells, and opened a new era in regenerative medicine.

## Shaping the Future

### Immune Modulatory Properties of Placenta MSC

Indeed, it is by merit of unique immune modulatory features, rather than differentiation, that placenta-derived MSC show promise for a wide range of regenerative medicine applications. Fast-forward to today there are over 20 clinical trials (excluding trials with unknown status) evaluating “placenta derived cells” and “placenta MSC” registered on the NIH Clinical Trials website (https://clinicaltrials.gov/) (Couto et al., 2017). The published or current clinical trials are either Phase I, II, or III and include a variety of inflammatory disorders, such as pulmonary idiopathic fibrosis (Chambers et al., 2014), peripheral artery disease, Crohn's disease (Mayer et al., 2013; Melmed et al., 2015), multiple sclerosis (Lublin et al., 2014), diabetes (Jiang et al., 2011), ischemic stroke, pulmonary sarcoidosis (Baughman et al., 2015), active rheumatoid arthritis, and muscle injury due to hip arthroplasty (Winkler et al., 2018).

There continues to be a significant advancement of our understanding in this field and many studies have shown that MSC from different regions of placenta can suppress the activation and modulate the function of various cells of the innate and adaptive immune systems, including macrophages, neutrophils, natural killer cells, dendritic cells, and T and B lymphocytes (Magatti et al., 2016). More specifically, many studies have shown that placental MSC can inhibit the proliferation of T lymphocytes, and can inhibit the differentiation into Th1 and Th17 while enhancing T regulatory cells. MSC can also promote the switch from a pro-inflammatory type 1 phenotype to an anti-inflammatory type 2 phenotype (Magatti et al., 2016). Several studies indicate that BM-MSC need to be “licensed” by inflammatory signaling to become fully immunosuppressive (Krampera et al., 2006; Ren et al., 2008; Sheng et al., 2008; Mougiakakos et al., 2011; Shi et al., 2012). In the case of hAMSC, priming by inflammatory cytokines is not a prerequisite for their immune-suppressive effects (Magatti et al., 2008; Rossi et al., 2012).

However, a word of caution is warranted since increasing experimental data indicate that hAMSC, similar to BM-MSC, can also stimulate immune cells both *in vitro* and *in vivo*. During fetal-maternal tolerance, amniotic cells could be involved in protecting the semiallogeneic fetus by two main threats; the first is the maternal immune system whereby suppression of immune response would be required, and the second is to protect against foreign and potentially dangerous pathogens which would require an enhanced immune response. One could imagine that this balance between immunosuppression and immunostimulation could explain the versatile immunomodulatory properties of hAMSC (Magatti et al., 2018). The mechanisms by which hAMSC and other placental MSC regulate the immune response and enable other cells to facilitate tissue repair during pathological processes remain under intense investigation.

### From MSC Differentiation to Paracrine/Endocrine Actions: A Focus on the MSC Secretome

It was once believed that to contribute in tissue regeneration MSC needed to be recruited to the site of tissue damage. However, in many cases there is low MSC engraftment and engrafted MSC tend to be short-lived, indicating the existence of other mechanisms by which MSC participate in regeneration (Vizoso et al., 2017). We now know with reasonable certainty that a significant part of the efficacy of MSC is mediated by their secreted factors. As a matter of fact, the success of MSC therapy in experimental models does not necessarily correlate with cell engraftment and replacement. Furthermore, inflammatory diseases have been successfully treated when using only the secretome of MSC. The recent recommendation by Arnold Caplan to rebrand MSC as “medicinal signaling cells” underlines the relevance of this shift in paradigm (Caplan, 2017).

Our group has significantly contributed to establishing that hAMSC can act via the release of bioactive mediators, *in vitro* by modulating immune cell proliferation and phenotype and *in vivo* by inducing therapeutic effects in immune-based disorders (Cargnoni et al., 2012, 2014; Rossi et al., 2012; Pianta et al., 2015; Pischiutta et al., 2016). However, the specific mediators and signaling pathways that affect the biology of adjacent and distant responder cells remain to be elucidated. The identification of these factors will shape the development of novel therapeutic strategies based on the secretome of hAMSC.

## Future Directions and New Prospects

It has been almost 2 decades since the discovery of MSC from fetal membranes of placenta, and the upcoming 2 decades of research will be animated by several debates and by answers to questions that the past and present research has posed.

For example, despite great strides on the understanding of MSC biology, the present is raising questions concerning a multitude of critical aspects for the clinical translation of MSC, regardless of their origin (Galipeau and Sensebe, 2018). Placental MSC face similar open questions and obstacles (Fierabracci et al., 2015), such as their economic sustainability and the determination of treatment dose, the latter being a basic medical question sometimes overlooked. As we move forward, it is essential to better understand the mechanisms by which these cells operate and to provide a solid basis so that placental MSC can be safely and efficiently used in patients. Given that we believe that amniotic cells contribute to tissue regeneration by promoting the resolution of inflammation, an improved understanding of the immune system's role in tissue regeneration will help in achieving this goal.

The future will also help develop and/or clarify several areas of research that have been less explored and/or are more debated. These include an understanding of the anti-microbial properties of placental MSC (a much less-explored but highly topical property), and the role of placental MSC in tumor progression. These will be paralleled with a more profound understanding of the cell secretome (e.g., preparation methods, bioactive factors, mechanisms of action), and the development of potency assays to predict clinical efficacy. In the following sections we will briefly touch upon these aspects.

### Anti-microbial Properties

An interesting yet often overlooked property of the amniotic membrane and cells derived thereof is their anti-microbial properties. Several groups have shown that amniotic MSC secrete antimicrobial factors both *in vitro* and *in vivo* (Kjaergaard et al., 1999, 2001; Buhimschi et al., 2004; Kheirkhah et al., 2012; Mao et al., 2017) and our lab has shown that MSC from the amniotic membrane can protect mice from experimental sepsis (Parolini et al., 2014). The antimicrobial properties of amniotic cells should be further investigated to understand how they could further foster a microenvironment favorable to regeneration.

### Pro- or Anti-tumor?

Besides their promise in regenerative medicine, placental MSC are also being studied for their potential applications as an anti-cancer strategy. As with MSC from other sources, placental MSC have been described to have dual actions, possessing both anti-tumor and pro-tumor properties (Silini et al., 2017a). The contradictions in these findings could be attributable to the variability and heterogeneity in MSC, to different isolation methods, passage number, or *in vitro* culture conditions, or to differences of the tumor cells, such as the tumor type or origin, degree of differentiation, the use of primary tumor cells or immortalized tumor cell lines. Preclinical studies are further confounded by differences in mouse models, xenogeneic or syngeneic tumor models, and dose, route, and timing of MSC administration. Important issues that remain to be determined before clinical use of placental MSC can be foreseen in the oncology field is the identity of the bioactive factors that contribute to the contrasting actions, their relevance over standard of care, and their potential combination with other anti-cancer therapies, such as target therapies or chemotherapy.

### Cells vs. Secretome

The secretome is the set of factors secreted by a cell into the extracellular space; these include free nucleic acids, soluble proteins, lipids, and extracellular vesicles (apoptotic bodies, microvesicles, and exosomes) (Raposo and Stoorvogel, 2013). The question to use cells or their secretome is complicated by the fact that the secretome of cells is specific and it changes in response to fluctuations in physiological states or pathological conditions and stimuli (Caplan and Correa, 2011). A minimal variation of cell culture conditions, such as phenotypic changes and senescence that may be observed during long term culture (Vono et al., 2018), influences the final MSC-products, including the MSC secretome. Thus, we should ask *which* secretome to use, or rather, which cell culture conditions should be used in order to obtain a “conditioned secretome” (Ferreira et al., 2018). The same holds true for cells, as the same MSC batches and production conditions are being used to treat different diseases such as graft-vs.-host disease, acute myocardial infarction, and diabetes. Thus, the MSCs are not fine-tuned for the disease being treated. One would hope to pretreat MSC in order to enhance the therapeutic response for the disease being treated (Ferreira et al., 2018), or to select MSC that possess an optimal response to the disease microenvironment.

The possibility of identifying the bioactive factors responsible for the therapeutic effect and thus translating them into medicine, is a high impact issue in current research. This will allow on one hand to identify those that are, putatively, the most relevant molecules involved in the immune modulatory effect and, on the other hand, to optimize the pharmacodynamics and pharmacokinetics of new drugs as well as allowing the delivery to the target cell. This approach will provide also important information on the mechanism of action of perinatal MSCs allowing for an understanding of how to exploit the endogenous properties of MSC cells in the maintenance of tissue homeostasis (Vizoso et al., 2017). In turn, the identification, synthesis, and GMP production of the bioactive factors could allow a highly standardized product that could also be tailored to the patient's needs. It could take many years to identify which are the bioactive molecules responsible for the therapeutic effects of perinatal MSCs (Baglio et al., 2012; Silini et al., 2013), despite the many advances in technology and analytical methodologies that are being developed.

We foresee that the choice between the use of hAMSC or the use of the conditioned secretome will largely depend on the clinical application and route of administration. If one takes into account the memory of the role of MSC within their original tissue to optimize use, this confers placental MSC a particular interest in immunomodulation and application in immune-based disorders (Silini et al., 2017b). Finding a common denominator to the different diseases will help in understanding the pathways targeted by hAMSC. In addition to the array of diseases in which hAMSC are being tested in clinical trials, the immunomodulatory properties of placental MSC could be prospected in the transplantation setting in order to support donor cell or tissue engraftment. Once again, the immune stimulatory capacities of hAMSC should be carefully considered in this possibility.

### Potency Assays

Another issue that should improve the successful application of amniotic MSC in regenerative medicine is the development of immunological potency assays (Galipeau and Krampera, 2015; Galipeau et al., 2016; Chinnadurai et al., 2018). These assays consist of *in vitro* tests to predict clinical response, and more specific to hAMSC, these tests would be crucial to predict both the immunosuppressive or immune-stimulation activity, and thus help guide the therapeutic application and potential efficacy.

Concerning the hAMSC secretome, standardization could be achieved by focusing on defining and identifying reproducible responses in potency assays based on standardized methods (i.e., standard operating procedures) for secretome production, rather than defining the secretome composition *per se* (Chinnadurai et al., 2018). This could in part override the difficult task of identifying the factors in the secretome and standardizing the secretome composition based on the cocktail of bioactive factors that exert the desired biological effect.

## Concluding Remarks

In the realm of regenerative medicine, the field of cellular therapy has gained more attention than any other field in biology and, furthermore, MSC represent important tools for the discovery of novel treatments with applications in regenerative medicine. We have learned that there are tremendous prospects of placental MSC in the field of regenerative medicine. However, there are significant issues that must be addressed in order to substantiate current studies and make these projections reality.

First, in the more wider setting of MSC, understanding which features are distinct for placental MSC and for other adult sources, such as bone marrow and adipose tissue, is relevant in understanding and optimizing clinical applications. There are major obstacles when trying to compare results from different MSC sources, such as heterogeneity in MSC populations and in experimental protocols. It remains to be verified if differences observed are due to isolation and culture methods of MSC (culture media, supplements, cell counts, passage number, etc.) or the readouts and stimuli used to determine cell characteristics. Standardization of assays for comparison and more comparative studies are necessary to give us a better understanding of distinct properties of placental MSC.

Second, MSC expansion protocols for the production of therapeutically active MSC and EV should be optimized (the same conditions do not necessarily apply). Manufacturing processes and release criteria are highly heterogeneous, and heterogeneity in the release criteria can result from an inaccurate definition of the MSC product and limited knowledge on the mechanisms of action. In all of this, therapeutic approaches must not only consider MSC donor variability, but also that of the MSC recipient, the latter of which also affects clinical outcome but is often neglected.

Third, the use of MSCs conditioned medium over cell-based applications has turned out to be an attractive opportunity. A cell-free therapeutic approach provides key advantages, such as resolving several safety concerns potentially associated with the transplantation of live and proliferative cells, it allows for storage without the use of potentially toxic cryopreservation agents and for easier production of a sufficient quantity of product (Vizoso et al., 2017). A detailed analysis on the culture conditions and on the selection of suitable cell sources evaluating the concentrations of bioactive factors and formulating a suitable dose, would substantiate the importance of the MSC secretome as a valid and promising approach in regenerative medicine (Gunawardena et al., 2019).

Fourth, a series of potency assays for cell-free products have to be developed in order to control their quality and quantity, and to predict their efficacy *in vivo* (Mohammadipoor et al., 2018). Finally, current challenges affecting the pharmaceutical industry must be considered, such as a shift toward older age groups resulting in increased chronic and degenerative disorders. In addition, a concerted effort is needed to facilitate access to innovation, meaning to provide favorable conditions for the translation of scientific advances into affordable therapies.

## Author Contributions

AS, AM, and AP drafted the work and OP critically revised and approved the work.

### Conflict of Interest Statement

The authors declare that the research was conducted in the absence of any commercial or financial relationships that could be construed as a potential conflict of interest.
